# Therapeutic Delivery of Phloretin by Mixed Emulsifier-Stabilized Nanoemulsion Alleviated Cerebral Ischemia/Reperfusion Injury

**DOI:** 10.3390/pharmaceutics17121599

**Published:** 2025-12-11

**Authors:** Tingting Huang, Changjing Wu, Wenchai Lu, Houbo Lv, Ronghui Jin, Jingyao Gan, Yuandong Zhang

**Affiliations:** 1 Department of Pharmacology, Key Laboratory of Basic Pharmacology of Guizhou Province, School of Pharmacy, Zunyi Medical University, Zunyi 563000, China; 2 Department of Immunology and Immunotherapy, Icahn School of Medicine at Mount Sinai, New York, NY 10029, USA

**Keywords:** phloretin, mixed emulsifiers, nanoemulsion, cerebral ischemia/reperfusion injury, oxidative stress

## Abstract

**Background:** Cerebral ischemia/reperfusion injury (CIRI) is a major challenge in ischemic stroke treatment. Phloretin (PHL), despite its potent antioxidant and anti-inflammatory properties, has limited clinical application due to poor oral bioavailability. This study aimed to develop an orally administered phloretin-loaded nanoemulsion (NE-PHL) to enhance brain delivery and neuroprotective efficacy against CIRI. **Methods:** NE-PHL was optimized via an orthogonal experimental design combined with ultrasonication. The optimized formulation was characterized for physicochemical properties and evaluated for pharmacokinetics and brain bioavailability. Its therapeutic efficacy was assessed in middle cerebral artery occlusion (MCAO) rats by measuring infarct volume, neurological scores, oxidative stress markers, and inflammatory cytokines. RNA sequencing analysis was performed to elucidate the underlying mechanisms. **Results:** The optimized NE-PHL exhibited a small droplet size (96.26 ± 0.86 nm), high encapsulation efficiency (84.58 ± 3.03%), and good storage stability over a period of 120 days. Pharmacokinetic studies showed a 2.72-fold increase in AUC _0–12h_ for NE-PHL compared to free PHL. In MCAO rats, NE-PHL treatment significantly improved neurological function, reduced cerebral infarct volume, attenuated oxidative stress, and modulated inflammatory responses by suppressing pro-inflammatory cytokines and enhancing anti-inflammatory activity. RNA sequencing analysis further confirmed coordinated downregulation of key pathways related to oxidative stress and inflammation. **Conclusions:** NE-PHL represents a promising oral nanotherapeutic strategy for the effective management of CIRI, offering enhanced bioavailability and significant neuroprotection.

## 1. Introduction

Stroke continues to represent a significant global health challenge, being the second leading cause of mortality and the foremost cause of long-term disability worldwide [1]. Etiologically, stroke is classified into ischemic stroke (IS) and hemorrhagic stroke, with IS accounting for 80–85% of all cases [2,3]. IS is typically precipitated by thrombosis or embolism, leading to an abrupt reduction in cerebral perfusion. Although reperfusion therapy is fundamental to clinical management, it paradoxically induces cerebral ischemia/reperfusion injury (CIRI), which exacerbates neuronal damage and contributes to irreversible neurological deficits [4,5,6]. The pathological mechanisms underlying CIRI include oxidative stress, neuroinflammation, and dysregulation of gene expression. Increasing evidence indicates that oxidative stress and neuroinflammation are key drivers of CIRI pathology [7]. Currently, the U.S. Food and Drug Administration (FDA) has not approved any agent that directly targets CIRI, highlighting the critical need for novel therapeutic interventions.

Phloretin (PHL), a natural dihydrochalcone flavonoid abundant in apples and pears, exhibits potent antioxidant activity. This property is attributed to its multiple phenolic hydroxyl groups, which facilitate the effective scavenging of harmful free radicals. Research has shown that PHL mitigates cisplatin-induced nephrotoxicity in mice by augmenting the activity of antioxidant enzymes while concurrently reducing lipid peroxidation markers [8]. In addition, PHL increased ileal antioxidant capacity by enhancing the total antioxidant capacity and superoxide dismutase activity [9]. Moreover, a recent study by Jyoti Chhimwal et al. demonstrated that PHL mitigated the progression of non-alcoholic fatty liver disease (NAFLD) via reducing oxidative damage and hepatic inflammation [10]. Importantly, pharmacological investigations have shown that PHL alleviates CIRI by activating the nuclear factor E2-related factor-2 (Nrf2) inflammation pathway [11]. Collectively, these findings underscore the potential of PHL as a promising antioxidant for CIRI, owing to its antioxidant and anti-inflammatory properties. Furthermore, PHL has shown neuroprotective potential in models of Alzheimer’s and Parkinson’s diseases by reducing oxidative stress, suppressing neuroinflammation, improving cholinergic function, and enhancing neuroplasticity [12,13,14], highlighting its potential as a therapeutic candidate for IS.

Although PHL demonstrates promising pharmacological activity in vivo, its therapeutic efficacy is limited by its unfavorable physicochemical and pharmacokinetic properties. Specifically, PHL exhibits extremely low aqueous solubility (~0.2 mM), poor oral absorption, and low bioavailability (~8.7%) [15]. Furthermore, PHL undergoes rapid metabolism and systemic clearance. In previous stroke model, therapeutic effects were achieved only via intraperitoneal injection of PHL dissolved in dimethyl sulfoxide at high doses (80 mg·kg^−1^·day^−1^ for 14 days) [11]. However, this regimen is clinically impractical due to toxicity concerns, including significant muscle loss and weakness following long-term administration [16]. These limitations emphasize the urgent need for advanced delivery strategies to improve the solubility, stability, and oral bioavailability of PHL. Simultaneously, existing studies have focused on the efficacy of PHL pretreatment. The potential protective effects of PHL with therapeutic administration remain unexplored.

To improve the poor aqueous solubility, limited oral absorption, and low bioavailability of PHL, various formulation strategies have been developed, among which nanoemulsion has attracted considerable attention. Nanoemulsions (NEs) offer multiple advantages, including high biocompatibility, low toxicity, substantial drug-loading capacity, and enhanced permeation across biological membranes [17]. Supporting oral, parenteral, transdermal, ocular, nasal, and pulmonary delivery, NEs can be compositionally tuned or surface-engineered (e.g., PEGylation, targeting ligands, charge modulation) to enable sustained/controlled release and active targeting, thereby improving efficacy while minimizing side effects [17,18]. These attributes are accelerating their transition from preclinical studies to commercialization, positioning NEs as a promising platform for safe, efficient pharmaceutical products.

Phospholipids are commonly employed as emulsifiers in nanoemulsion formulations due to their natural origin and favorable safety profile, making them preferable to synthetic surfactants. Lecithin (LEC), in particular, can self-assemble at the oil–water interface and form a viscoelastic stabilizing film via hydrogen bonding [19]. Nevertheless, the zwitterionic nature of most phospholipids restricts their ability to generate sufficient electrostatic or steric repulsion between droplets in oil-in-water (O/W) emulsions, thereby promoting droplet aggregation [20]. To enhance stability and prevent coalescence, supplementary emulsifiers are often incorporated into LEC-based systems.

In pharmaceutical formulations, surfactants from the Kolliphor series are widely used, partly due to their ability to inhibit P-glycoprotein activity, an effect that can enhance drug oral bioavailability and thus increase therapeutic efficacy [21]. These surfactants are approved by FDA for both oral and intravenous administration [22]. For example, Kolliphor^®^ HS15 comprises a lipophilic 12-hydroxystearic acid moiety and a hydrophilic polyethylene glycol chain; this amphiphilic architecture facilitates efficient encapsulation of poorly soluble drug molecules [23]. Similarly, Kolliphor^®^ RH40 has also been employed as a solubilizer and successfully used in self-emulsifying drug delivery systems (SEDDS), including formulations containing squalene (SQU) [24]. Building on this application, SQU was therefore selected as the oil phase in the present study owing to its exceptional biocompatibility and intrinsic bioactivity [25,26,27], with the aim of synergistically enhancing the performance of phloretin-loaded nanoemulsion (NE-PHL).

The present study aimed to develop NE-PHL by combining the stabilizing capacity of LEC, the amphiphilic versatility of Kolliphor^®^ surfactants, and the bioactive properties of SQU, with the goal of enhancing the oral bioavailability and brain tissue distribution of PHL. The nanoemulsion was prepared via probe ultrasonication-induced disruption of emulsion droplets, and the emulsifier content was optimized using orthogonal design experiments. Its physicochemical characteristics, storage stability, and in vitro release profile were systematically evaluated, followed by comprehensive in vivo assessments of pharmacokinetics and bio-distribution. The therapeutic efficacy was further investigated in an MCAO rat model, assessing multiple outcome parameters. To the best of our knowledge, this is the first report on the use of NE-PHL for post-stroke treatment targeting CIRI. This multifunctional nanoemulsion design may offer a versatile delivery platform for other therapeutic agents with poor aqueous solubility.

## 2. Materials and Methods

### 2.1. Materials

PHL (purity >98%) was obtained from Shanghai Adamas Reagent Co., Ltd. (Shanghai, China). SQU (purity ≥98%) was purchased from Sigma-Aldrich (St. Louis, MO, USA). Egg yolk-derived LEC was sourced from AVT Medical Technology Co., Ltd. (Shanghai, China). Kolliphor^®^ RH40 was obtained from Dulai Biotechnology Co., Ltd. (Nanjing, China). Kolliphor^®^ HS15 was purchased from MedChemExpress (MCE, Monmouth Junction, NJ, USA). Tetrahydrofuran (THF) was purchased from Shanghai McLean Biochemical Technology Co., Ltd. (Shanghai, China). 1,1’-dioctadecyl-3,3,3’,3’-tetramethylindodicarbocyanine, 4-chlorobenzenesulfonate salt (DID) was obtained from MeilunBio (Dalian, China). 2,3,5-triphenyltetrazolium chloride (TTC) was obtained from Solarbio (Beijing, China). HPLC-grade methanol and acetonitrile were obtained from Shanghai Adamas Reagent Co., Ltd. (Shanghai, China). Enzyme-linked immunosorbent assay (ELISA) kits for reactive oxygen species (ROS), hydrogen peroxide (H_2_O_2_), malondialdehyde (MDA), superoxide dismutase (SOD), glutathione (GSH), glutathione peroxidase (GSH-Px), interleukin-6 (IL-6), interleukin-1 beta (IL-1β), interleukin-4 (IL-4), interleukin-10 (IL-10), and tumor necrosis factor-alpha (TNF-α) were purchased from Renjie Biotechnology Co., Ltd. (Shanghai, China). Ultrapure water was used in all experiments.

### 2.2. Preparation of NE-PHL

NE-PHL was fabricated with an optimized ultrasonication method [28]. Briefly, PHL was first dissolved in THF. Concurrently, SQU, LEC, and Kolliphor^®^ RH40 were weighed into a vessel. The PHL solution was then introduced into the mixture containing the excipients and stirred until a homogeneous solution was obtained. Subsequently, Kolliphor^®^ HS15 was added dropwise under constant stirring to form a primary emulsion. The primary emulsion was ultrasonically homogenized at 130 W for 5 min using a probe sonicator. Finally, the organic solvent was evaporated under reduced pressure using a rotary evaporator (120 rpm, 10 min), yielding the final NE-PHL formulation.

### 2.3. Characterization

#### 2.3.1. Morphological Observation

For morphological examination, the NE-PHL was initially diluted (1:5, *v*/*v*) to an appropriate concentration. Approximately 20 μL of sample was deposited onto a 400-mesh carbon-coated copper grid and allowed to dry at room temperature, then inverted onto 2% phosphotungstic acid for 1–2 min. Excess stain was removed with filter paper. The sample was then observed using a transmission electron microscope (JEM-1400FLASH, JEOL, Tokyo, Japan).

#### 2.3.2. Droplet Size, Size Distribution, and Zeta Potential

The mean droplet size, polydispersity index (PDI), and zeta potential of the NE-PHL were measured using a Malvern Zetasizer Nano ZS90 instrument (Malvern Instruments Ltd., UK). Prior to measurement, each sample was diluted 1:500 with ultrapure water and transferred into a clear disposable zeta cell. All measurements were conducted at 25 °C.

#### 2.3.3. Encapsulation Efficiency and Drug Loading

To determine the encapsulation efficiency and drug loading, the nanoemulsion was treated with a solubilizing agent composed of anhydrous ethanol and THF (6:5, *v*/*v*) to release the encapsulated drug. After centrifugation (12,000 rpm, 10 min), the drug content in the supernatant was quantified by high-performance liquid chromatography–ultraviolet (HPLC-UV), as described in Supporting Information Section S2.1. The encapsulation efficiency and drug loading were calculated using the following equations:

(1) $$\mathrm{Encapsulation}\;\mathrm{Efficiency}(\%)=\frac{M_{\mathrm{loaded}\;\mathrm{PHL}}}{M_{\mathrm{total}\;\mathrm{PHL}}}\times 100\%$$

(2) $$\mathrm{Drug}\;\mathrm{Loading}(\%)=\frac{M_{\mathrm{loaded}\;\mathrm{PHL}}}{M_{\mathrm{excipients}}+M_{\mathrm{total}\;\mathrm{PHL}}}\times 100\%$$

M_total PHL_ denotes the total mass of PHL utilized in the formulation, M_loaded PHL_ refers to the mass of PHL encapsulated within the NEs, and M_excipients_ represents the total mass of excipients present in the system.

#### 2.3.4. Stability Evaluation

The nanoemulsion was stored under two conditions (ambient temperature and 4 °C). Under both conditions, samples were collected at predetermined intervals to measure the mean droplet size and PDI on days 1, 3, 5, 7, 10, 15, 20, 30, 60, 90, and 120, and the encapsulation efficiency on days 1, 7, 15, 30, 60, 90 and 120. These measurements were used to evaluate the physical stability of the formulation during storage. Additionally, we evaluated the stability of the nanoemulsion in simulated gastrointestinal fluids; detailed methods are provided in Section S2.3 of the Supplementary Materials.

#### 2.3.5. Drug Release in Vitro

The in vitro release profiles of PHL and NE-PHL were evaluated using a dialysis method. Briefly, each sample was placed in a dialysis bag (MWCO 8000 Da, Repligen) and immersed in phosphate-buffered saline (PBS, pH 7.4), simulated gastric fluid (SGF, pH 1.2), and simulated intestinal fluid (SIF, pH 6.8). All release media were prepared in accordance with the methods specified in the Chinese Pharmacopeia 2025 (for details, see Supporting Information, Section S2.2). The system was incubated at 37 °C with continuous agitation (120 rpm/min). Aliquots (1 mL) of the release medium were collected at predetermined time points (1, 2, 3, 4, 6, 8, 10, 12, 24, 36, and 48 h). An equal volume of fresh pre-warmed medium was immediately added after each sampling to maintain sink conditions throughout the study. Cumulative drug release was analyzed by HPLC-UV.

### 2.4. Pharmacokinetic Studies

#### 2.4.1. Animals

Healthy male Sprague Dawley (SD) rats, weighing about 250 ± 20 g, were obtained from Hunan Slake Kingda Laboratory Animal Co., Ltd. (Changsha, China). The certificate number is SCXK-Xiang-2019-0004. The rats were kept at a steady temperature of 23 °C. They had a 12 h light and dark cycle and could eat and drink freely. All procedures followed standard guidelines for animal care and were approved by the Ethics Committee for Animal Experiments of Zunyi Medical University. The protocol number is (2021)2-599, which was approved on 18 December 2021.

#### 2.4.2. Pharmacokinetic Protocol

A pharmacokinetic study was carried out on SD rats, which were randomly assigned to two groups receiving either PHL or the NE-PHL formulation. Drawing on previous pharmacokinetic data [15], each rat was given a single oral dose of 100 mg·kg^−1^ PHL. Blood samples were collected from the orbital venous plexus into heparinized tubes at specific time points (0.17, 0.33, 0.5, 0.75, 1, 1.5, 2, 3, 4, 6, 8, and 12 h). Plasma was separated by centrifugation at 8000 rpm for 10 min (4 °C). For sample preparation, 55 μL of plasma was combined with 10 μL of a methanolic taxifolin solution (12.04 ng/mL, internal standard) and 200 μL of methanol. The mixture was vortexed and then centrifuged at 12,000 rpm for 10 min. The resulting supernatant was analyzed using ultra-performance liquid chromatography–tandem mass spectrometry (UPLC–MS/MS) method (for details, see Supporting Information, Section S2.4).

#### 2.4.3. Tissue Distribution

To prepare NE-DID, PHL was replaced by DID as the active compound. DID was first dissolved in THF. Separately, SQU, LEC, and Kolliphor^®^ RH40 were weighed into a glass vessel. The DID solution was subsequently added to the excipient mixture and stirred until a uniform solution was obtained. An aqueous phase containing 0.5% (*w*/*v*) Kolliphor^®^ HS15 was then added dropwise under constant stirring to form a primary emulsion. The primary emulsion was ultrasonically homogenized at 130 W for 5 min using a probe sonicator. The organic solvent was removed under reduced pressure using a rotary evaporator (120 rpm, 10 min), yielding the final NE-DID formulation. SD rats were randomly allocated into three groups: control group, free DID, and NE-DID. The rats were euthanized via cervical dislocation at 3 h and 6 h following oral administration. The hearts, livers, spleens, lungs, kidneys, and brains were harvested, rinsed with saline, blotted dry with filter paper, and subsequently imaged using a small animal in vivo optical imaging system (Berthold Technologies, Bad Wildbad, Germany).

### 2.5. Pharmacological Studies

#### 2.5.1. Ischemic Stroke Induction—MCAO Model

Focal CIRI was induced using the MCAO model [29]. Rats were anesthetized with 2% pentobarbital sodium (30 mg/kg, i.p.) and maintained under isoflurane on a heating pad. A midline neck incision was performed to expose the left common carotid artery (CCA), external carotid artery (ECA), and internal carotid artery (ICA). Following ligation of the CCA bifurcation and temporary clamping of the CCA and ICA, a small incision was made in the ECA stump, and a silicone-coated monofilament was advanced approximately 1.8 cm into the ICA to occlude the middle cerebral artery. In the sham group, the arteries were exposed without filament insertion. After 2 h of ischemia, the filament was withdrawn to allow reperfusion. Successful MCAO was confirmed by laser Doppler flowmetry (Moor Instruments Ltd., Axminster, UK).

#### 2.5.2. Groups and Treatment

Two hours after occlusion, rats were randomly assigned to five groups: Sham (surgery without MCAO, no treatment), MCAO (0.9% NaCl), MCAO + SQU (5 mg/kg SQU), MCAO + PHL (5 mg/kg PHL), and MCAO + NE-PHL (PHL-loaded NE equivalent to 5 mg/kg PHL). Treatments were administered orally 4 h after reperfusion and continued twice daily for 3 days.

#### 2.5.3. Behavioral Testing

Neurological function was assessed using the Longa 5-point scoring system after 3 days of NE-PHL treatment. As previously described [30], rats were suspended by the tail approximately 35 cm above the ground to observe forelimb posture. Subsequently, they were placed on a flat surface and gently pushed at the shoulder to assess asymmetric resistance, followed by an assessment of their walking behavior as they moved freely. Neurological deficits were graded on a scale from 0 to 4, where 0 stands for no deficit, 1 refers to mild deficit (failure to fully extend the contralateral forepaw), 2 means moderate deficit (circling to the contralateral side), 3 represents severe deficit (falling to the contralateral side), and 4 equals absence of spontaneous walking or loss of consciousness (Table S2).

#### 2.5.4. TTC Staining

The volume of cerebral infarction was assessed using TTC staining. MCAO Rats were euthanized, and their brains were promptly extracted, chilled on ice, and cleared of the cerebellum and olfactory bulb. The brains were rinsed with saline, blotted dry, and frozen at −20 °C for 20 min. Coronal sections were incubated in a 0.4% TTC solution at 37 °C for 60 min, with gentle inversion every 10 min. The sections were then fixed in 4% formaldehyde for 48 h at 4 °C, and imaged. All brain tissue sections from each rat were weighed, followed by the isolation and weighing of the infarcted brain tissue portions. The infarct volume (%) was calculated using the formula below:

(3) $$\mathrm{Infart}\;\mathrm{volume}\left(\%\right)=\frac{\mathrm{Weight}\;\mathrm{of}\;\mathrm{infarcted}\;\mathrm{brain}}{\mathrm{Weight}\;\mathrm{of}\;\mathrm{infarcted}\;\mathrm{brain}+\mathrm{Weight}\;\mathrm{of}\;\mathrm{normal}\;\mathrm{brain}}\times 100\%$$

#### 2.5.5. Cerebral Oxidative Stress and Neuroinflammation Determination

Following MCAO, all animals were administered treatment for three days post-reperfusion and subsequently euthanized via cervical dislocation. Brain tissues from the ischemic penumbra were promptly dissected on ice, homogenized with a ratio of 10 mg tissue per 30 µL PBS, and centrifuged at 3000 rpm for 10 min. The supernatants were collected and stored at −80 °C for subsequent analysis of inflammatory and oxidative markers using ELISA.

### 2.6. RNA Sequencing Analysis

RNA Sequencing transcriptome profiling was conducted on ischemic cerebral cortex tissues from the following experimental groups: MCAO, MCAO+PHL, and MCAO+NE-PHL. Sequencing was executed by LC-Bio Technology Co., Ltd. (Hangzhou, China). Gene expression levels were quantified by calculating the fragments per kilobase of the exon model per million mapped fragments (FPKM) for each sample. Genes exhibiting a *p*-value <0.05 and a fold change >2 were classified as differentially expressed genes (DEGs). Advanced volcano plots, heatmaps, and Kyoto Encyclopedia of Genes and Genomes (KEGG) analyses were generated using the Oebiotech online tool (https://www.bioinformatics.com). Venn diagrams were produced using the OmicStudio online tool (http://www.omicstudio.cn/tool/, accessed on 15 December 2024). KEGG enrichment pathways were visualized through the application of Cytoscape software (version 3.7.1).

### 2.7. Preliminary Safety Evaluation in Vivo

A short-term safety assessment was performed to evaluate potential adverse effects following administration. Rats were administered the drug treatment for three consecutive days. On day 5, animals were euthanized, and the whole blood was collected from the abdominal aorta. Whole blood analysis was conducted using a veterinary automated hematology analyzer (Mindray BC-2800Vet, Shenzhen, China) to determine the levels of red blood cells, white blood cells, monocytes, neutrophils, lymphocytes, and platelets. Major organs, including the heart, liver, spleen, lungs, kidneys, and intestines, were harvested and fixed in 4% paraformaldehyde. The tissues were embedded in paraffin, sectioned, stained with hematoxylin and eosin (H&E), and examined by light microscopy (Nikon TE2000, Tokyo, Japan) for observation of pathological changes.

### 2.8. Statistical Analysis

Data analysis was conducted utilizing GraphPad Prism software (version 9.0). The findings are expressed as mean ± standard deviation (SD). Group comparisons were performed using ANOVA, followed by Dunnett’s multiple comparisons test for multiple comparisons, or unpaired two-tailed Student’s t-tests where applicable. A *p*-value of less than 0.05 was deemed statistically significant.

## 3. Results

### 3.1. Optimized NE-PHL Formulation

An orthogonal experimental design was utilized to identify the optimal formulation compositions (Table S1). Emulsions with varying prescription ratios were prepared, and their droplet size, PDI, and zeta potential were measured using dynamic light scattering. As summarized in Table 1, formulations with the lowest oil phase content or highest emulsifier content (F3, F7, F8, F9) exhibited gradual bottom precipitation and distinct phase separation. Emulsions with LEC to Kolliphor^®^ RH40 ratios of 1:2 and 1:3 had optimal droplet size and PDI. Based on comprehensive K-value analysis, PDI < 0.3, the optimal formulation was determined as A2B3C3, consisting of SQU (100 mg), Kolliphor^®^ RH40 (90 mg), LEC (30 mg), and Kolliphor^®^ HS15 (0.5%).

**Figure 1:**
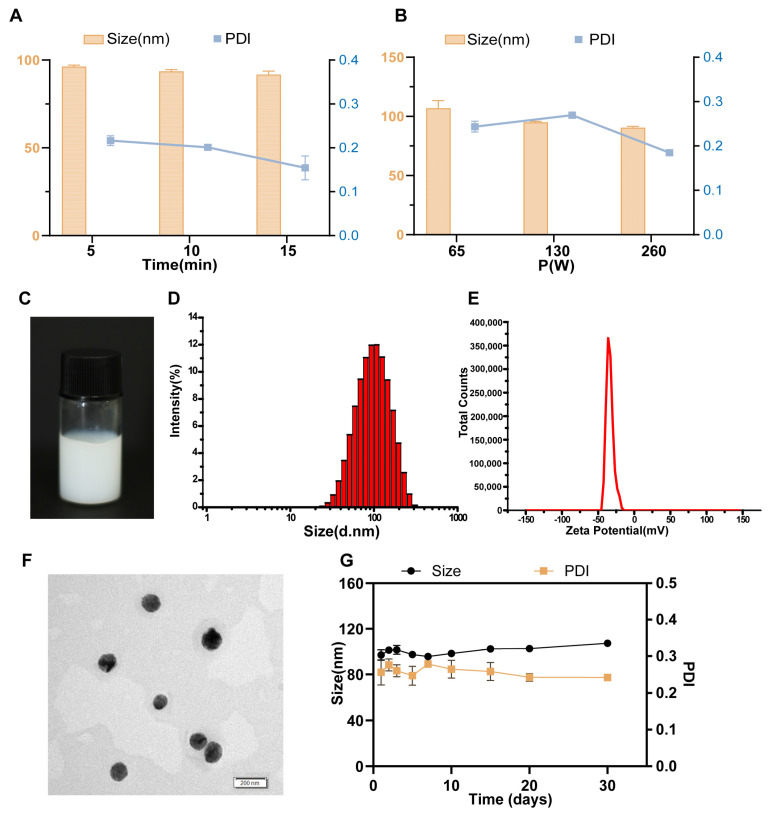
Characterization of NE-PHL. Optimization on the ultrasonic times (**A**) and ultrasonic powers (**B**) of NE-PHL. (**C**) Macroscopic appearance of the optimized NE-PHL. The size (**D**) and zeta potential (**E**) of NE-PHL. (**F**) TEM image of NE-PHL. (**G**) Changes in the average size and PDI of NE-PHL during 30-day storage at 4 °C. Data are presented as mean ± SD (n = 3).

Subsequent optimization of processing parameters revealed that sonicating for 5 min gave a droplet size of 96.26 ± 0.86 nm. Longer sonication did not significantly reduce it further (Figure 1A). Likewise, sonication at 130 W produced droplets of 94.89 ± 0.92 nm, and higher power did not substantially improve the size (Figure 1B). Therefore, the optimal conditions were set at 5 min and 130 W.

### 3.2. Characterization

The optimized formulation is presented as a homogeneous milky suspension, exhibiting characteristic opalescence upon dilution (Figure 1C). The droplet size was 97.01 ± 4.61 nm, with a PDI of 0.26 ± 0.03 and a zeta potential of −39.26 ± 0.23 mV (Figure 1D,E). Transmission electron microscopy revealed spherical droplets with a relatively uniform size distribution (Figure 1F). The formulation demonstrated an encapsulation efficiency of 84.58 ± 3.03% and a drug loading capacity of 4.32 ± 0.62%. After being stored at 4 °C for one month, droplet size and PDI of NE-PHL showed no significant alterations (Figure 1G), which confirmed the absence of drug precipitation and phase separation. Importantly, the nanoemulsion retained consistent droplet size, PDI, and satisfactory encapsulation efficiency under long-term (four-month) storage at both 25 °C (Table S3) and 4 °C (Table S4), thereby demonstrating the long-term storage stability of the optimized nanoemulsion formulation.

Figure S2 describes the stability of NE-PHL in SGF and SIF. In SGF, droplet size decreased markedly within the first 2 h, then gradually increased, approaching the initial value by 8 h. The PDI rose sharply above 0.3 at 2 h before declining to near baseline levels at 8 h (Figure S2A). In SIF, both droplet size and PDI showed no significant change throughout the incubation (Figure S2B). This suggests that, rather than being disrupted, the droplets may undergo aggregation in the intestinal environment. Overall, NE-PHL displays reduced colloidal stability in simulated gastrointestinal fluids.

### 3.3. Drug Release in Vitro

**Figure 2:**
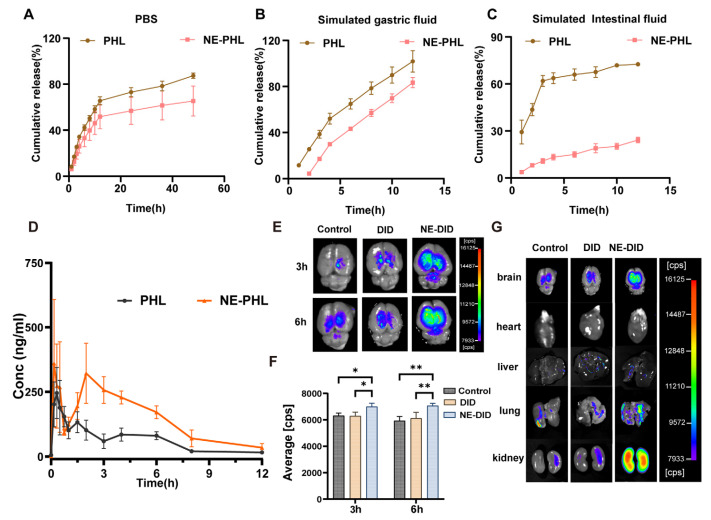
Results of the in vitro drug release and in vivo pharmacokinetics of NE-PHL. The in vitro release profile of NE-PHL in PBS (**A**), in SGF (**B**), and in SIF (**C**) (*n* = 3). (**D**) The concentration–time curves of free PHL and NE-PHL after oral administration in rats. (**E**–**F**) Qualitative and quantitative analysis of the fluorescence signals of NE-PHL in brain tissue at 3 h and 6 h following oral administration. (**G**) Bio-distribution of the fluorescence signal of NE-PHL 6 h after oral administration in rats at 6 h. Data are expressed as mean ± SD (*n* = 6). * *p* < 0.05, ** *p* < 0.01 compared to the control group.

The release profiles of NE-PHL and PHL are shown in Figure 2. In PBS, PHL reached a cumulative release of 65.53 ± 3.52% at 16 h and nearly 87.39 ± 2.34% at 48 h. In contrast, NE-PHL exhibited a slower release, with only 51.71 ± 10.61% and 65.32 ± 13.07% released at the respective time points (Figure 2A). In SGF, PHL was completely released within 12 h, whereas 83.39 ± 4.41% of NE-PHL was released (Figure 2B). In SIF, PHL showed a rapid release of 63.66 ± 3.51% within 4 h, followed by a plateau. NE-PHL, however, demonstrated sustained-release characteristics, with only 24.16 ± 1.79% released over 12 h (Figure 2C).

These results indicate that NE-PHL possesses sustained-release properties. The drug is released sequentially from the internal oil phase, through the surfactant interfacial film, and into the aqueous phase. The higher release rate in gastric fluid suggests partial destabilization of nanoemulsion under acidic conditions. Complete release was not observed in other media, which is likely due to the retention of lipid components by the semi-permeable membrane. While this may lead to an underestimation of the absolute release, it does not affect the comparative analysis of the release behavior.

### 3.4. NE-PHL Increased PHL Levels in Blood and Brain of Rats

Based on UPLC–MS/MS analysis (Figure S1), the plasma concentration–time profile of the drug was established (Figure 2D). The corresponding pharmacokinetic parameters are summarized in Table 2. Following oral administration at 100 mg/kg, the NE-PHL formulation exhibited a 2.72-fold increase in AUC _0–12h_, a higher C_max_, and a reduced CLz/F compared with the PHL solution. The short half-life of PHL suggests rapid distribution and metabolism. Combined with the in vitro findings, these results indicate that the improved oral bioavailability of NE-PHL arises from its enhanced stability and sustained-release characteristics. The improvement likely results from reduced enzymatic degradation and improved mucus penetration, consistent with mechanisms previously reported for nanoemulsion systems [31].

Fluorescence imaging has confirmed an enhanced uptake and retention of NE-PHL in the brain, with more pronounced signals detected between 3 and 6 h post-administration compared to PHL (Figure 2E–G). These findings indicate that NE-PHL facilitates accelerated absorption, increases plasma concentrations, and enhances brain penetration, thereby supporting its potential to augment the pharmacodynamic effects of PHL in CIRI.

### 3.5. Pharmacodynamic Study

#### 3.5.1. NE-PHL Alleviated Neurological Dysfunction in CIRI Rats

**Figure 3:**
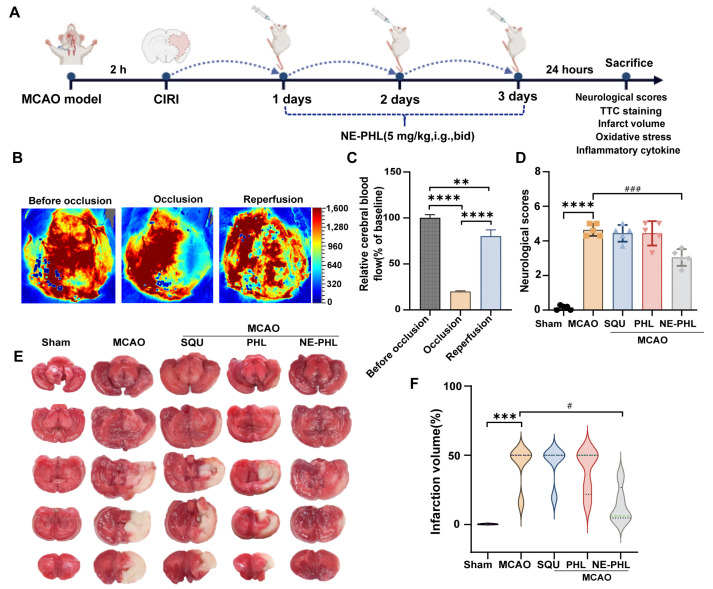
Protective effects of NE-PHL on CIRI following oral administration. (**A**) Schematic representation of the experimental design for NE-PHL treatment in MCAO Rats; (**B**) Laser scatter imaging for monitoring alterations in CBF; (**C**) Semi-quantitative analysis of CBF. Data are presented as mean ± SD (*n* = 5). ** *p* < 0.01, **** *p* < 0.0001; (**D**) Neurological deficit scores of all experimental groups; (**E**) Representative images of TTC-stained brain slices; (**F**) Analysis of cerebral infarct volume. Data are presented as mean ± SD (*n* = 5). *** *p* < 0.001, **** *p* < 0.0001 compared to the sham group; ^#^ *p* < 0.05, ^###^ *p* < 0.001 compared to the MCAO group. The dash lines from up to down indicate 75th percentile, median line and 25th percentile.

The therapeutic efficacy of NE-PHL was assessed in a rat model of MCAO (Figure 3A). Laser speckle contrast imaging verified the successful induction of CIRI, as evidenced by a reduction in cerebral blood flow (CBF) to approximately 20% of baseline during ischemia, with recovery to approximately 80% following reperfusion (Figure 3B–C). Neurological function was evaluated using the Longa scoring method after three consecutive days of drug administration post-reperfusion. Preliminary experiments confirmed that, under the same conditions, the blank nanoemulsion did not significantly improve neurological function or reduce cerebral infarct volume (Figure S3). The MCAO group demonstrated significant deficits (4.64 ± 0.35, *p* < 0.0001 vs. sham). Treatments with SQU and PHL resulted in minimal improvement (4.44 ± 0.48 and 4.44 ± 0.70, respectively), whereas NE-PHL markedly enhanced motor coordination, reducing the neurological score to 3.04 ± 0.48 (*p* = 0.0002 vs. MCAO group) (Figure 3D). These findings suggest that oral administration of NE-PHL effectively mitigates neurological deficits and facilitates functional recovery following CIRI.

#### 3.5.2. NE-PHL Decreased the Infarct Volume in CIRI Rats

TTC staining showed that the infarcted tissue appeared as unstained areas, whereas viable brain tissue was stained red. No infarction was detected in the sham group, while extensive lesions were observed in the MCAO group. Treatment with SQU, PHL, or NE-PHL reduced infarct size to varying degrees, as indicated by increased red staining area in the affected hemisphere (Figure 3E). Quantitative analysis, as illustrated in Figure 3F, showed that the infarction volume in the MCAO group reached 43.16 ± 15.29%. Treatment with SQU and PHL produced minimal reductions (43.05 ± 13.43% and 38.75 ± 16.08%, respectively), whereas NE-PHL treatment significantly decreased infarct volume to 13.85 ± 13.16%.

#### 3.5.3. NE-PHL Attenuated the MCAO-Induced Oxidative Stress

**Figure 4:**
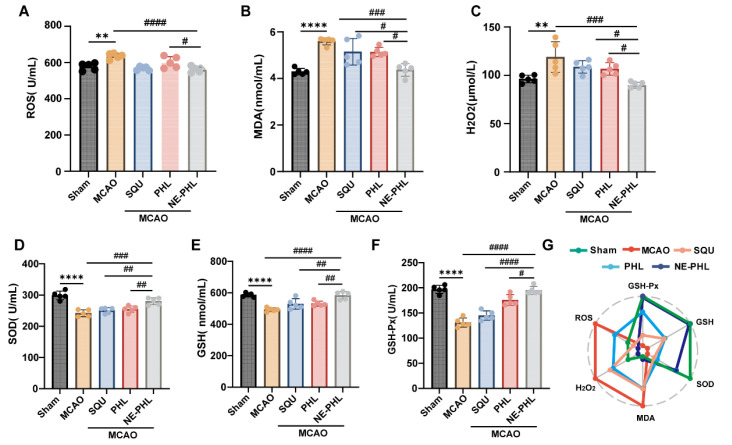
NE-PHL mitigated oxidative stress in the cerebral tissue of rats with CIRI. The activities of oxidative stress markers were quantified, including (**A**) ROS, (**B**) MDA, (**C**) H_2_O_2_, (**D**) SOD, (**E**) GSH, and (**F**) GSH-Px (*n* = 5). (**G**) Radar chart analysis of the effects of NE-PHL on oxidative stress markers. Data are presented as mean ± SD ** *p* < 0.01, **** *p* < 0.0001 compared to the sham group; ^#^ *p* < 0.05, ^##^ *p* < 0.01, ^###^ *p* < 0.001, ^####^ *p* < 0.0001 compared to the NE-PHL group.

To assess the antioxidant potential of NE-PHL, the activities of SOD, GSH, GSH-Px, MDA, H_2_O_2_, and ROS were quantified using ELISA. As shown in Figure 4, markers of oxidative stress were significantly altered in the MCAO group compared to the sham group, as evidenced by the following: ROS (*p* = 0.0014), MDA (*p* < 0.0001), H_2_O_2_ (*p* = 0.0035). Post-stroke administration of NE-PHL attenuated these elevations, reducing the enzyme activities of ROS (*p* < 0.0001), MDA (*p* < 0.0001), and H_2_O_2_ (*p* = 0.0002) relative to the MCAO group. But PHL and SQU produced no significant effects (Figure 4A–C). Conversely, the activities of the antioxidant enzymes SOD, GSH, and GSH-Px were reduced in the MCAO group. Neither PHL nor SQU treatment improved these parameters. Whereas the antioxidant enzyme activity was restored to near-sham levels through post-stroke administration of NE-PHL (MCAO + NE-PHL group vs. MCAO group, SOD: *p* = 0.0002; GSH: *p* < 0.0001; GSH-Px: *p* < 0.0001) (Figure 4D–F). Analysis of oxidative stress markers via radar charts revealed a pathological alteration in the MCAO group, which was counteracted by NE-PHL treatment, restoring a near-normal profile. In contrast, PHL and SQU demonstrated minimal therapeutic efficacy. (Figure 4G).

Oxidative stress is a key contributor to the pathophysiology of CIRI. During aerobic respiration, mitochondria are responsible for the production of ATP and small quantities of ROS, which are typically neutralized by antioxidant enzymes such as SOD. In IS, the depletion of oxygen results in a metabolic shift towards glycolysis, leading to the accumulation of lactic acid, acidosis, and an overproduction of ROS. This overwhelms the antioxidant defenses, resulting in oxidative damage, mitochondrial dysfunction, and neuronal death. Consequently, antioxidant therapies are crucial, with a focus on inhibiting ROS production, scavenging free radicals, and enhancing ROS degradation [33]. Our results suggested that NE-PHL exerted superior antioxidant activity compared with PHL. The protective effect was associated with a reduction in oxidative damage markers (MDA, H_2_O_2_, ROS) and restoration of antioxidant enzymes (SOD, GSH, GSH-Px).

#### 3.5.4. NE-PHL Modulated Inflammatory Cytokine Release

**Figure 5:**
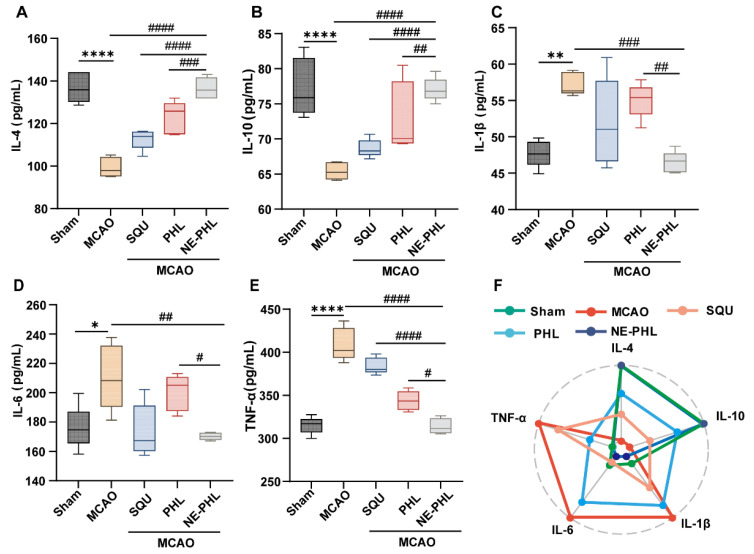
Post-stroke administration of NE-PHL notably reduced neuroinflammation in the cerebral tissue of MCAO rats. Levels of (**A**) IL-4, (**B**) IL-10, (**C**) IL-1β, (**D**) IL-6, and (**E**) TNF-α were quantified using ELISA (*n* = 5). (**F**) Radar chart analysis of the effects of NE-PHL on inflammatory cytokines. Data are presented as mean ± SD. * *p* < 0.05, ** *p* < 0.01, **** *p* < 0.0001 compared to the sham group; ^#^ *p* < 0.05, ^##^ *p* < 0.01, ^###^ *p* < 0.001, ^####^ *p* < 0.0001 compared to the NE-PHL group.

Excessive neuroinflammation is a key driver in CIRI. ELISA results confirmed a pronounced inflammatory imbalance in the MCAO group, characterized by a significant reduction in anti-inflammatory cytokines (IL-4, IL-10) and a concurrent marked increase in pro-inflammatory cytokines (IL-1β, IL-6, TNF-α) compared to the sham group (Figure 5). Nevertheless, NE-PHL treatment effectively reversed this imbalance, significantly elevating anti-inflammatory cytokine levels (Figure 5A–B: IL-4: *p* < 0.0001; IL-10: *p* < 0.0001) and suppressing pro-inflammatory mediators (Figure 5C–E: IL-1β: *p* = 0.0003; IL-6: *p* = 0.0041; TNF-α: *p* < 0.0001) relative to the MCAO group. In contrast, neither PHL nor SQU showed significant effects. Radar chart analysis further visualized that the cytokine profile in the MCAO group was essentially inverted compared to the sham group. NE-PHL treatment restored this profile to close-sham levels, while PHL and SQU produced only minor corrections (Figure 5F).

### 3.6. RNA Sequencing Analysis of NE-PHL in CIRI Rats

**Figure 6:**
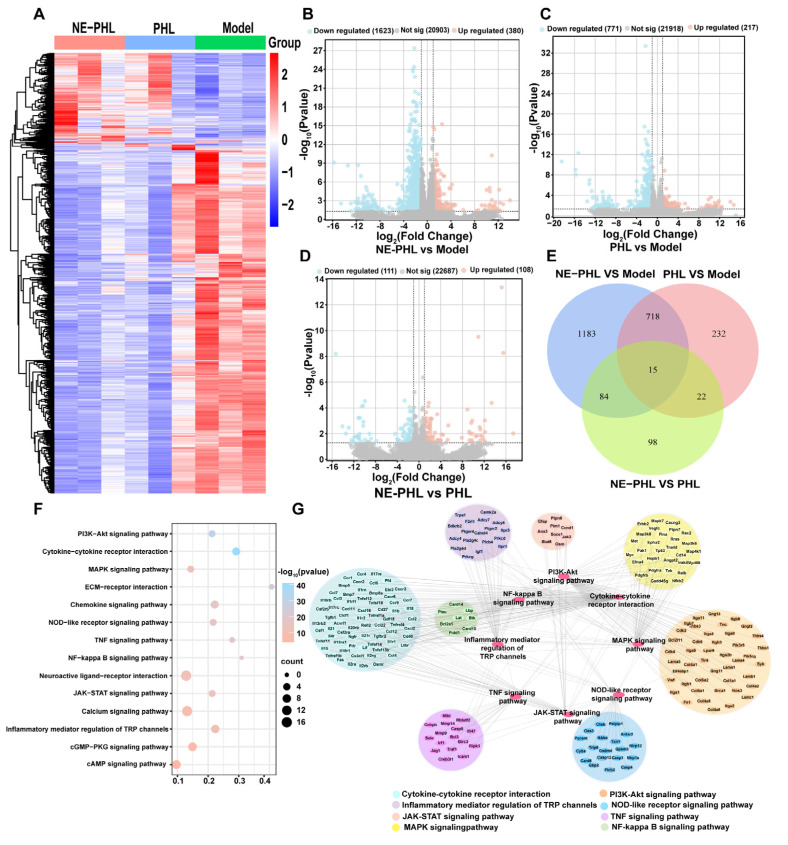
RNA sequencing analysis of differential gene expression. (**A**) Heatmap showing correlated mRNA expression across MCAO, PHL, and NE-PHL groups. (**B**–**D**) Volcano plots of DEGs for NE-PHL vs. MCAO (**B**), PHL vs. MCAO (**C**), and NE-PHL vs. PHL (**D**) comparisons. The dashed line separates upregulated genes from downregulated genes. (**E**) Venn diagram. (**F**) KEGG pathway analysis of DEGs between MCAO and NE-PHL groups. (**G**) KEGG pathway enrichment network visualized with Cytoscape 3.7.1. (*n* = 3).

To investigate the mechanisms by which NE-PHL mitigates CIRI, RNA sequencing was performed on brain tissues from MCAO rats after three days of treatment. Unsupervised clustering of RNA-seq data from three biological replicates per group (MCAO, PHL, and NE-PHL) revealed distinct transcriptional profiles. A heatmap of DEGs showed marked transcriptional differences between the NE-PHL and MCAO groups (Figure 6A). Comparing NE-PHL with the MCAO controls, volcano plot analysis identified 2003 significantly altered genes (380 upregulated and 1623 downregulated; Figure 6B). Comparison between PHL and MCAO yielded 988 altered genes (217 upregulated, 771 downregulated; Figure 6C), whereas a direct comparison of NE-PHL with PHL resulted in 219 DEGs (108 upregulated, 111 downregulated; Figure 6D). A Venn diagram further revealed that NE-PHL induced substantially more unique transcriptional changes, with 1183 non-overlapping DEGs specific to the NE-PHL versus MCAO comparison (Figure 6E). Incorporating drugs into nanoemulsions enables simultaneous modulation of multiple mechanisms, thereby enhancing therapeutic efficacy.

Functional enrichment analysis of downregulated DEGs (MCAO group vs. MCAO + NE-PHL) revealed significant suppression of biological processes associated with oxidative stress and inflammation, such as “nucleotide-binding and oligomerization domain (NOD)-like receptor signaling pathway”, “nuclear factor-kappa B (NF-κB) signaling pathway”, “mitogen-activated protein kinase (MAPK) signaling pathway”, “phosphatidylinositol 3’-kinase (PI3K)-Akt signaling pathway”, and “calcium signaling pathway”, among others (Figure 6F). A pathway enrichment network was subsequently constructed using Cytoscape (Figure 6G), which identified key regulators, including MYD88, NF-κB2, Toll-like receptor 4, RELB proto-oncogene, interleukin-1, and tumor necrosis factor family, as central nodes in most of the suppressed pathways.

### 3.7. In Vivo Biosafety of NE-PHL

**Figure 7:**
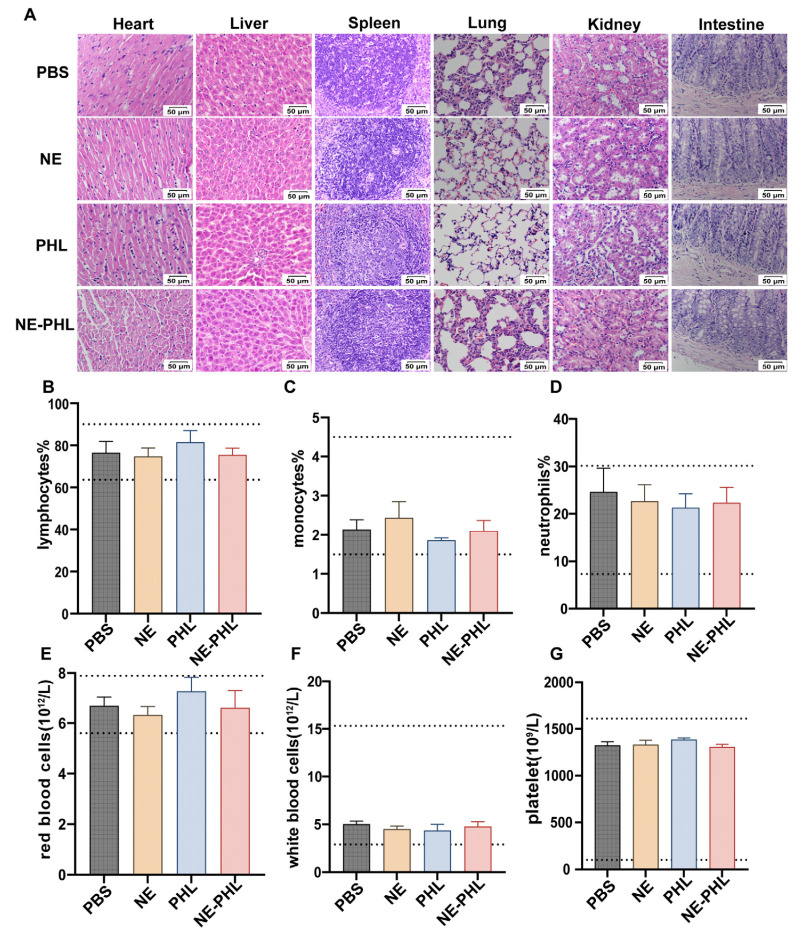
In vivo biosafety of NE-PHL. (**A**) H&E staining of the major organs of rat. Scale bar: 50 μm. Frequency of lymphocytes (**B**), monocytes (**C**), neutrophils (**D**), red blood cells (**E**), white blood cells (**F**), and platelets (**G**) determined in peripheral blood using a veterinary autohematology analyzer. Data are presented as mean ± SD (*n* = 7). Dashed lines stand for the normal level range of lymphocytes, monocytes, neutrophils, red blood cells, white blood cells and platelet in blood.

Finally, the in vivo biocompatibility and safety of NE-PHL were systematically evaluated. On day 5 after administration, major organs (heart, liver, spleen, lung, kidney, and intestine) were harvested from rats and examined by H&E staining. Histological analyses showed no detectable structural abnormalities in the NE-PHL group compared with the PBS control (Figure 7A). To further evaluate the systemic safety, complete blood count (CBC) assays were conducted. No significant changes were observed among populations in peripheral blood, including lymphocytes, monocytes, neutrophils, red blood cells, white blood cells, and platelets (Figure 7B–G). Together, these findings indicate that NE-PHL exhibits a favorable biosafety profile, underscoring its suitability for biomedical applications.

## 4. Discussion

Various delivery systems, including hyaluronic acid-modified nanoparticles [34], biodegradable polymeric nanocomposites [35] and mixed micelles [36], have been investigated as carriers for PHL. These approaches have demonstrated multiple pharmacological benefits, such as enhancing antioxidant activity, facilitating recovery from myocardial ischemia, reducing hepatic fibrosis, and slowing the progression of non-alcoholic fatty liver disease [37]. The restrictive permeability of the blood–brain barrier (BBB) constitutes a major obstacle to the effective delivery of neuroprotective agents, posing a significant challenge to the success of pharmacological interventions. To date, orally administrable nanoformulations of PHL tailored for IS therapy remain scarcely reported. In this study, we developed an orally administrable NE-PHL to overcome the poor solubility and low bioavailability of PHL. The optimized formulation displayed favorable physicochemical characteristics (Figure 1), accelerated absorption, and a pronounced increase in brain tissue distribution compared with free PHL (Figure 2). In MCAO rat model, therapeutic administration of NE-PHL markedly alleviated CIRI (Figure 3).

Previous studies have shown that PHL pretreatment activates the Nrf2/ARE pathway in CIRI models, significantly reducing oxidative neuronal damage [11]. Nrf2, a critical regulator of the antioxidant response, activates cytoprotective genes encoding enzymes such as SOD and catalase [38]. Consistent with these findings, our results indicate that NE-PHL is highly effective in mitigating oxidative stress-associated neuronal injury (Figure 4).

Importantly, NE-PHL exerts neuroprotective effects not only by suppressing pro-inflammatory pathways but also by actively promoting anti-inflammatory cytokine secretion. The anti-inflammatory activity of PHL has been associated with MAPK activation, which stimulates macrophage autophagy and drives a phenotypic shift toward anti-inflammatory states, thus attenuating neuroinflammation [39] (Figure 5). Furthermore, PHL has been reported to modulate other immune cell populations, including inhibition of T-cell and dendritic-cell activation in vitro [40]. Transcriptomic analysis in the present work revealed that the neuroprotective properties of NE-PHL were mediated by coordinated downregulation of key oxidative stress- and inflammation-related signaling pathways (Figure 6). These results are consistent with the research findings, collectively demonstrating that NE-PHL confers neuroprotection against CIRI primarily through modulation of oxidative and inflammatory responses within the brain microenvironment. Nevertheless, the complex regulatory networks underlying these processes, particularly across diverse cellular populations, warrant further investigation.

A notable distinction between our study and prior stroke models lies in dosing strategy. Earlier work required high-dose intraperitoneal injections of PHL (80 mg·kg^−1^·day^−1^ for 14 days) dissolved in dimethyl sulfoxide [10] to achieve therapeutic benefit. In contrast, our formulation achieved comparable—or even superior—anti-CIRI efficacy with a substantially lower dose (10 mg·kg^−1^·day^−1^) administered orally over just three days. Pharmacokinetic evaluation demonstrated that NE-PHL achieved superior exposure and brain distribution compared to free PHL at the same dose (Figure 2D). This improvement reflects more efficient delivery, enabling significant dose reduction, which may ultimately enhance patient compliance and improve the overall treatment experience. Although the present study demonstrates that NE-PHL effectively enhances oral bioavailability, improves brain tissue distribution, and confers robust neuroprotection in a CIRI injury model, several limitations should be acknowledged. First, the current findings are based solely on short-term animal experiments using MCAO rats; comprehensive evaluations of long-term efficacy and safety are needed to firmly establish therapeutic applicability. Second, although transcriptomic analysis provided valuable insights into the modulation of oxidative stress and inflammation-related pathways, the precise cellular targets and upstream regulatory mechanisms remain undefined. In particular, the identification of specific immune cell subsets and elucidation of their dynamic interactions within the post-ischemic brain microenvironment warrant further mechanistic investigation.

## 5. Conclusions

An orally delivered nanoemulsion was prepared in this study to overcome the poor solubility and bioavailability limitations of PHL. The optimized NE-PHL formulation demonstrated not only favorable physicochemical characteristics and robust stability but also a distinctive sustained-release profile. Pharmacokinetic studies revealed accelerated absorption and a marked increase in brain distribution compared to PHL. NE-PHL led to significant recovery of neurological function, reduced cerebral infarct size, attenuated oxidative damage, and modulation of neuroinflammatory responses in MCAO model rats. RNA sequencing analysis further indicated that the neuroprotective efficacy was mediated via coordinated downregulation of key signaling pathways involved in oxidative damage and inflammation. Importantly, the therapeutic benefit was evident even at low doses, suggesting potential for dose reduction, improved patient compliance, and enhanced safety. Together, these results potentiate NE-PHL as an oral nanotherapeutic candidate for CIRI and lay a translational foundation for the clinical application of PHL-based nanomedicines.

## Figures and Tables

**Table 1:** Results and range analysis of orthogonal experiment. Slash refers to precipitation occurred on day 3.

Formulation	Factor			Particle Size (nm)	PDI	Zeta (mV)	EE%
	A	B	C				
F1	1	1	1	96.69	0.046	−35.42	78.36
F2	1	2	2	114.13	0.530	−42.31	82.38
F3	1	3	3	96.04	0.210	−33.14	/
F4	2	1	2	55.48	0.233	−33.93	84.02
F5	2	2	3	68.93	0.260	−35.31	78.51
F6	2	3	1	66.84	0.330	−22.12	85.22
F7	3	1	3	86.21	0.193	−33.00	/
F8	3	2	1	126.27	0.230	−36.34	/
F9	3	3	2	17.43	0.227	−18.00	/
K1	102.29	79.46	96.60				
K2	63.75	103.11	62.35				
K3	76.64	60.10	83.73				
R	38.54	43.01	34.25				

**Table 2:** Pharmacokinetic parameters of PHL and NE-PHL after oral administration in rats (*n* = 6).

Pharmacokinetic Parameters	PHL (100 mg·kg^−1^)	NE-PHL (100 mg·kg^−1^)
AUC 0–12 h (h × ng/mL)	686.50 ± 198.19	1867.84 ± 692.02 *
AUC 0–∞ (h × ng/mL)	761.63 ± 191.83	2033.383 ± 726.77 **
MRT (0–12 h) (h)	3.10 ± 1.09	4.20 ± 1.01
MRT (0–∞) (h)	4.77 ± 1.69	5.282 ± 2.157
t1/2z (h)	2.02 ± 0.88	2.60 ± 1.31
T max (h)	1.37 ± 1.54	1.87 ± 1.70
CLz/F (L/h/kg)	2.40 ± 50.88	0.90 ± 16.92 **
C_max_ (ng/mL)	302.09 ± 144.25	581.28 ± 401.86
F	100%	272.08%

AUC _0–12h_: area under the concentration–time curve from time 0–12 h; MRT_(0–12h)_: mean residence time from 0 to 12 h; t1/2z: half-life calculated based on the terminal elimination rate constant; T _max_: time at C_max_; CLz/F: the apparent volume of bodily fluids from which a drug is cleared per unit time; C_max_: maximum plasma concentration; F: relative oral bioavailability(%) = [AUC (test)/AUC (control)] × [Dose(control)/Dose (test)] × 100% [32]. Data are shown as the means ± SD (*n* = 6). * *p* < 0.05; ** *p* < 0.01; compared with the PHL group.

## Data Availability

The original contributions presented in this study are included in the article/Supplementary Material. Further inquiries can be directed to the corresponding authors.

## Supplementary Materials

The following supporting information can be downloaded at https://www.mdpi.com/article/10.3390/pharmaceutics17121599/s1, Figure S1: Mass Spectrometry Analysis Results; Figure S2: In vitro stability assessment of NE-PHL in SGF and SIF; Figure S3: Blank NE treatment in a middle cerebral artery occlusion (MCAO) model to evaluate its effects on cerebral ischemia/reperfusion injury after oral administration. Table S1: Factors and levels of *L*_9_*^(^3^3)^* orthogonal test; Table S2: Longa 5 levels scoring principle; Table S3: Results of long-term testing (25 ± 2 °C); Table S4: Results of long-term testing (4 ± 2 °C)
