# Supplementary material for: Therapeutic Delivery of Phloretin by Mixed Emulsifier-Stabilized Nanoemulsion Alleviated Cerebral Ischemia/Reperfusion Injury
Source: Pharmaceutics. 2025 Dec 11;17(12):1599. doi: 10.3390/pharmaceutics17121599 (PMC12737318; doi:10.3390/pharmaceutics17121599)
Supplement: Supplementary file 1 [file pharmaceutics-17-01599-s001.zip › pharmaceutics-3985340-supplementary.pdf]

pharmaceutics

MDPI

Supplementary Materials

Therapeutic Delivery of Phloretin by Mixed Emulsifiers-Stabilized Nanoemulsion Alleviates Cerebral Ischemia/Reperfusion Injury

Tingting Huang, Changjing Wu, Wenchai Lu, Houbo Lv, Ronghui Jin and Yuandong Zhang

S2. Materials and Methods

S2.1. High-Performance Liquid Chromatographic Conditions

High-performance liquid chromatography (HPLC-UV) analysis was performed on a Waters 2695 system (Waters, Milford, MA, USA) equipped with a Kromasil 1005-C18 column (4.6 × 250 mm, 5 µm). The mobile phase composed of ultrapure water and acetonitrile (50:50, v/v), was delivered at a flow rate of 1.0 mL/min. Detection was carried out using a UV detector set at 280 nm, and the column temperature was maintained at 35°C.

S2.2. Preparation of Simulated Gastric and Intestinal Fluids

S2.2.1. Preparation of Simulated Gastric Fluid (SGF)

Simulated gastric fluid (SGF) was prepared by dissolving 10 g of pepsin in 800 mL of purified water. Then, 16.4 mL of dilute hydrochloric acid was added under vigorous stirring until complete dissolution. The pH of the solution was adjusted to 1.2 using dilute HCl or NaOH solutions as required. Finally, the volume was made up to 1000 mL with purified water.

S2.2.2. Preparation of Simulated Intestinal Fluid (SIF)

Simulated intestinal fluid (SIF) was prepared by dissolving 6.8 g of monopotassium phosphate (KH_2PO_4) in 500 mL of purified water under continuous stirring. The pH of the resulting solution was adjusted to 6.8 using 0.1 mol/L sodium hydroxide solution, followed by the addition of 10 g of trypsin with stirring until complete dissolution. The mixture was then transferred to a 1000 mL volumetric flask and diluted to volume with purified water to obtain the final solution.

S2.3. In Vitro Stability Assessment of NE-PHL in SGF and SIF

NE-PHL was mixed with SGF (pH 1.2) or SIF (pH 6.8) at a 1:50 (v/v) ratio and incubated on an orbital shaker at 37°C with continuous agitation (120 rpm). Samples were collected at predefined time points (0, 0.5, 1, 2, 4, 6, 8, 10, and 24 h) and the average particle size and polydispersity index (PDI) were determined using a Zetasizer Nano ZS90 (Malvern Instruments Ltd., UK).

S2.4. Mass Spectrometric Conditions

The concentration of PHL in plasma was determined using a UPLC-MS/MS system coupled to a QqQ Quantum triple quadrupole mass spectrometer with an electrospray ionization (ESI) source (Waters, Milford, MA, USA). Chromatographic separation was achieved on a Waters ACQUITY UPLC BEH C18 column (2.1 mm × 50 mm, 1.7 µm) main-

 https://doi.org/10.3390/pharmaceutics17121599

tained at 40°C. The mobile phase consisted of 0.1% formic acid in water (A) and acetonitrile (B), eluted at a flow rate of 0.3 mL/min with the following gradient program: 0–2.5 min, 85% A to 60% A; 2.5–3.0 min, 60% A to 15% A; 3.0–3.1 min, 15% A to 5% A; 3.1–4.0 min, 5% A; 4.0–4.1 min, 5% A to 85% A; and 4.1–5.5 min, 85% A. The injection volume was 2 μL.

Mass spectrometric detection was performed in negative ion mode using multiple reaction monitoring (MRM). The ion source temperature was set at 150°C, with a desolvation temperature of 500°C. The capillary and cone voltages were 3.0 kV and 24 V, respectively, with a collision energy of 16 eV. The desolvation and cone gas flows were 75 $L \cdot h^{-1}$ and 150 $L \cdot h^{-1}$, respectively. The deprotonated precursor-to-product ion transitions monitored for quantification were m/z 273.1 for phloretin and m/z 303.1 for the internal standard (taxifolin).

S2.5. Optimize the Process by $L_9(3^3)$ Orthogonal Experimental Design

Table S1. Factors and levels of $L_9(3^{3)}$ orthogonal test.

Level	Factor		
	**SQU (mg) /A**	**LEC:Kolliphor® RH40 /B**	**Kolliphor® HS15 (%) /C**
1	50	1:1	0.1
2	100	1:2	0.25
3	150	1:3	0.5

S2.6. Neurological Performance

Table S2. Longa 5 levels scoring principle.

Level	Criteria
0	Proper function
1	Left forelimb unable to extend
2	Turning to the left
3	Leaning to the left during gait
4	Paralysis
5	Death

S3. Results and discussion

S3.1. Mass Spectrometry Analysis Results

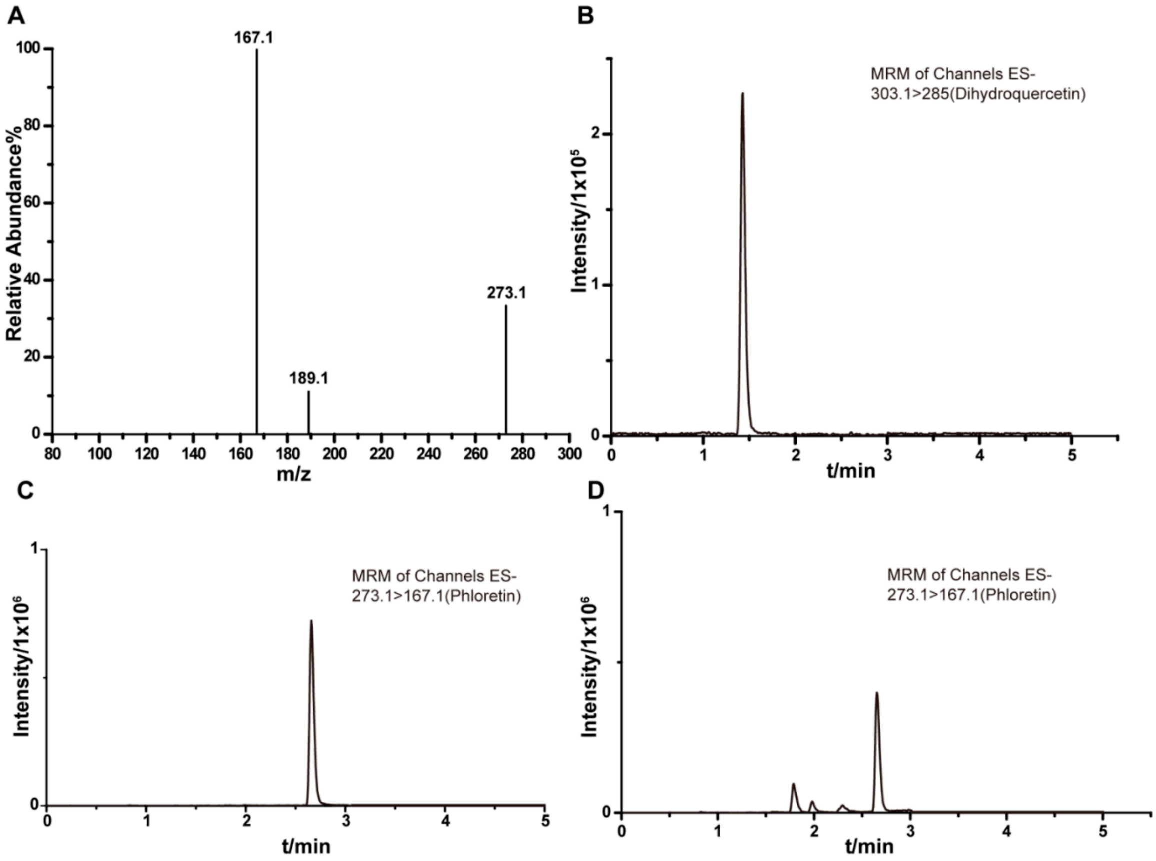

Figure S1. Mass Spectrometry Analysis Results. (**A**) High-resolution ESI mass spectrum of phloretin (PHL). The observed $[M-H]^-$ ion at m/z 273.1 corresponds to the molecular formula $C_{15}H_{14}O_5$ (calcd for $C_{15}H_{14}O_5$, 273.1). (**B-D**) Representative extracted ion chromatograms (EICs) for (**B**) the internal standard (taxifolin), (**C**) the phloretin standard, and (**D**) phloretin detected in a plasma sample.

S3.2. Storage Stability Test Results

Table S3. Results of long-term testing (25 ± 2°C).

Time (days)	Appearance	Particle size (nm)	PDI	Zeta potential (mV)	EE%
1	**No drug leakage**	97.01±4.61	0.26±0.03	-39.26±0.23	87.42±1.25
3	**No drug leakage**	101.58±3.89	0.26±0.02	-38.30±0.78	/
5	**No drug leakage**	97.36±2.95	0.25±0.03	-38.64±0.56	/
7	**No drug leakage**	95.63±1.81	0.28±0.00	-38.25±0.19	87.32±0.34
10	**No drug leakage**	98.33±1.45	0.26±0.02	-38.03±0.03	/
15	**No drug leakage**	102.31±1.87	0.25±0.02	-38.57±1.23	87.12±2.40
20	**No drug leakage**	102.58±2.00	0.24±0.01	-38.52±0.27	/

30	**No drug leakage**	105.20±3.20	0.26±0.03	-38.44±0.69	87.01±3.62
60	**No drug leakage**	107.19±1.96	0.24±0.00	-38.23±0.19	86.78±4.55
90	**No drug leakage**	107.66±1.59	0.24±0.01	-38.66±0.73	86.63±2.51
120	**No drug leakage**	108.33±2.60	0.24±0.01	-38.25±0.64	86.47±1.27

Table S4. Results of long-term testing (4 ± 2°C).

Time (days)	Appearance	Particle size (nm)	PDI	Zeta potential (mV)	EE%
1	**No drug leakage**	101.34±2.81	0.27±0.02	-38.51±0.22	88.17±0.56
3	**No drug leakage**	101.44±1.67	0.26±0.00	-38.75±3.75	/
5	**No drug leakage**	98.41±1.59	0.26±0.02	-38.50±2.54	/
7	**No drug leakage**	100.94±0.32	0.23±0.00	-39.42±0.81	88.23±1.48
10	**No drug leakage**	101.07±2.58	0.25±0.01	-38.54±1.42	/
15	**No drug leakage**	101.41±1.38	0.27±0.00	-37.53±1.67	87.58±1.14
20	**No drug leakage**	102.69±1.60	0.23±0.00	-38.36±2.74	/
30	**No drug leakage**	102.74±1.46	0.25±0.00	-38.41±1.12	87.69±2.31
60	**No stratification**	101.18±0.51	0.27±0.00	-39.57±0.58	86.58±1.50
90	**No drug leakage**	105.00±1.94	0.22±0.01	-38.60±0.27	86.84±0.73
120	**No drug leakage**	103.26±1.07	0.25±0.01	-40.16±0.32	86.39±2.67

S3.4. In Vitro Stability Assessment of NE-PHL in SGF and SIF

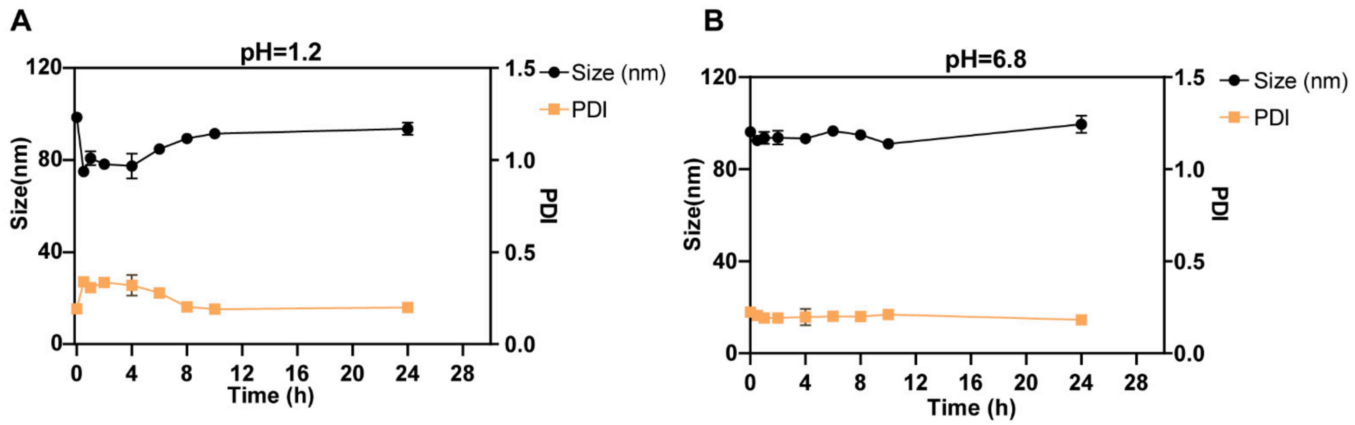

Figure S2. *In vitro* stability assessment of NE-PHL in SGF and SIF. (A) Variation in particle size and PDI of NE-PHL over 24 h of incubation with SGF. (B) Variation in particle size and PDI of NE-PHL over 24 h of incubation with SIF.

S3.5. Effects of Blank NE on CIRI Following oral Administration

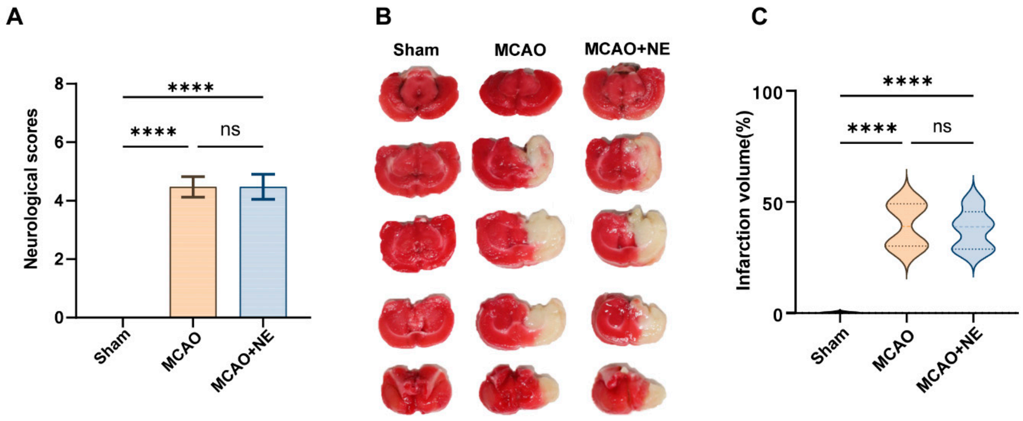

Figure S3. Blank NE treatment in a middle cerebral artery occlusion (MCAO) model to evaluate its effects on cerebral ischemia/reperfusion injury after oral administration. (A) Neurological deficit scores for the sham, MCAO, and blank NE groups. (B) Representative TTC-stained brain slices. (C) Quantitative analysis of cerebral infarct volume. Data are expressed as mean ± SD (n = 5). ****p < 0.0001 vs. sham group; ns: not significant. The dash lines from up to down indicate 75th percentile, median line and 25th percentile.
